# A SIMPLE ANIMAL MODEL OF EXERCISE REVEALS A MOLECULAR DETERMINANT OF LONG TERM HEALTH MAINTENANCE

**DOI:** 10.1093/geroni/igac059.1070

**Published:** 2022-12-20

**Authors:** Monica Driscoll

**Affiliations:** Rutgers, The State University of New Jersey, New Brunswick, New Jersey, United States

## Abstract

At all stages of life, both dynamic gene expression changes and events that “lock in” particular programs that promote health and maintenance are critical factors in aging trajectories. We have a long-term interest in the fundamental biology of healthy maintenance, a topic that has led us to consider multiple facets of healthspan. A powerful whole-organism intervention with maintenance-promoting, anti-disease, anti-aging impact is exercise. The molecular and cellular mechanisms that mediate long-term system-wide exercise benefits, however, remain poorly understood, especially as applies to “off target” tissues that do not participate directly in training activity. We are investigating the basic biology of exercise benefits using the simple 959-celled model C. elegans. We found that multiple daily swim sessions are essential for exercise adaptation, leading to enhanced expression of muscle structural genes and improved locomotory performance. Importantly, swim exercise training enhances whole-animal health parameters such as mitochondrial respiration and mid-life survival, increases functional healthspan of pharynx and intestine, and enhances nervous system health by increasing learning ability of adults and protecting against neurodegeneration in models of tauopathy, Alzheimer’s disease, and Huntington’s disease. Remarkably, swim training only during early adulthood induces long-lasting systemic benefits that in several cases are still detectable well into mid-life. Our investigation of the molecular requirements for long-term maintenance (“legacy effects”) revealed that deletion of the sole C. elegans extracellular superoxide dismutase gene SOD-4 changes this pattern. sod-4 mutants are able to swim train to gain measurable benefits, but do not maintain the healthspan improvements that wild type animals do, defining extracellular SOD-4 as a powerful mid-life maintenance factor associated with exercise experience. Our talk will discuss transcriptomic analysis of exercise and SOD-4, as well as our current understanding of the molecular mechanisms operative. Notably, mammalian extracellular SOD ecSOD has been shown to promote exercise associated health and protection against oxidative stress insults (PMID: 32220789 Yan, Spaulding 2020), implicating ecSOD in a conserved role for health maintenance and underscoring potential for therapeutic translation.

